# Highly Efficient Spin-Current Operation in a Cu Nano-Ring

**DOI:** 10.1038/srep37398

**Published:** 2016-11-21

**Authors:** Benedict A. Murphy, Andrew J. Vick, Marjan Samiepour, Atsufumi Hirohata

**Affiliations:** 1 Department of Physics, University of York, Heslington, York YO10 5DD, United Kingdom; 2 Department of Electronics, University of York, Heslington, York YO10 5DD, United Kingdom

## Abstract

An all-metal lateral spin-valve structure has been fabricated with a medial Copper nano-ring to split the diffusive spin-current path. We have demonstrated significant modulation of the non-local signal by the application of a magnetic field gradient across the nano-ring, which is up to 30% more efficient than the conventional Hanle configuration at room temperature. This was achieved by passing a dc current through a current-carrying bar to provide a locally induced Ampère field. We have shown that in this manner a lateral spin-valve gains an additional functionality in the form of three-terminal gate operation for future spintronic logic.

In order to maintain the current miniaturisation trends in nanoelectronic devices, the spin degree of freedom in the electron has attracted a great deal of attention^1^,^2^. Here, spin-polarised electrons (or holes) can be used as a carrier of information and can be either processed in a three-terminal transistor or stored as the magnetisation of a ferromagnetic layer in a memory cell. For the former transistor-type configuration, it was not long after the first observation of spin-polarised electron injection into a non-magnetic Au layer^3^ that three-terminal devices were proposed^4^. Examples of three-terminal operation have been largely restricted to semiconductor systems where gate operation is provided by manipulating electron spins by using electro-magnetic waves^5^,^6^,^7^. In particular, spin-polarised light emitting diodes have been widely used to investigate spin injection in semiconductors^5^,^6^. However, semiconductor systems require a micron scale path to manipulate their spins. On the other hand, all-metal systems have attracted great interest due to their nanometric dimensions^8^,^9^ since the work by Jedema *et al.*^10^. Generally, a ferromagnetic metal is used to polarise an electrical charge current as it passes into a non-magnetic metallic wire. The current is drawn off in one direction, yet the induced spin imbalance diffuses away in all directions in accordance with Fick’s law and the transit of the spin imbalance may be detected by a second ferromagnetic wire at a distance determined by the material-dependent spin-diffusion length. Thus, one may observe the diffusion of a spin imbalance in the absence of any net flow of charge.

In order to realise a spin-current logic cell using such a non-local lateral spin-valve (LSV) geometry, the operation of the spin-current is the next step to be achieved. Spin accumulation can be controlled by injecting spin-polarised electrons from two ferromagnetic nano-wires, which are aligned with an angle of 45° for both sides of the Cu nano-wire^11^, which establishes that spin currents can be summed by dual injection to provide a spin-current logic circuit. In this letter, we have modified a Cu nano-wire into a nano-ring in order to split the spin-current path into two. The spin current is operated upon by the application of potential to a current carrying bar that generates an Ampère field. One of the two channels of the nano-ring experiences a larger perpendicular Ampère field, changing the phase of the spin current and gives rise to gate functionality in a spin-current three-terminal device. We observe an efficient spin-current modulation, which is found to be up to 30% more effective than the conventional simple Hanle effect^3^, suggesting the arithmetical operability of the spin currents in this manner.

## Results

**Figure 1:**
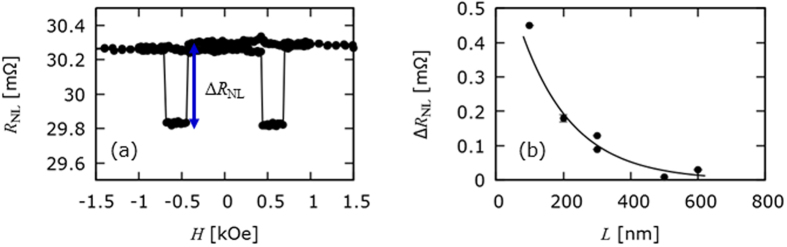
Spin operator. (**a**) Typical non-local measurement from a lateral spin-valve and (**b**), the decay in the corresponding non-local resistance with increased distance between the injector and detector (filled circles) with least-squares fit to Equation (1) (black line).

Two sets of samples were fabricated: a set of conventional lateral spin-valve devices and a set of lateral spin-valves with a ring patterned into the spin channel between the ferromagnetic injector and detector wires. The interfacial resistances between the Ni_80_Fe_20_ and the Cu spin diffusion channel were measured and found to be in the range 0.93 to 6.29 Ω • μm^2^, where the variance in resistance was assumed to be due to residue from the lift-off process. The devices displayed the characteristic non-local resistance behavior as shown in Fig. 1(a) and the conductivity of the Cu was found to be 3.76 × 10^7^ S/m. All measurements were taken at room temperature.

The conventional lateral spin-valve devices were used to identify the spin diffusion length in high purity Cu. The length of the spin diffusion channel (measured from the centre of the Ni_80_Fe_20_/Cu interfaces) was varied between 200 and 700 nm and the non-local voltage, induced by the application of a pulsed current at the injector interface, due to the spin imbalance at the detector interface was measured as shown in Fig. 1(b).

The electron spin imbalance diffuses away from the injection point in a manner that can be described a solution in the form of a Bloch equation^12^. The junctions, although of high resistance, are Ohmic and so fall into the intermediate limit, yielding a solution of the form:


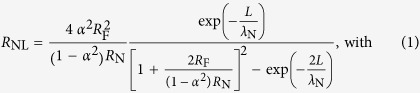



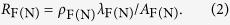


where +(−)*R*_NL_ is the magnitude of the non-local resistance in the parallel (anti-parallel) injector/detector magnetisation alignment, *R*_F(N)_ is the spin resistance of the ferromagnetic (non-magnetic) channel, *α* is the spin-polarisation of the injected electrons, *L* is the separation between the injector/detector, *ρ*_F(N)_ is the resistivity of the ferromagnetic (non-magnetic) wire, *λ*_F(N)_ is the spin diffusion length in the magnetic (non-magnetic) wire and *A* is the cross-sectional area of the ferromagnetic (non-magnetic) wire. Now, in the presence of a magnetic field, the spin precession, *λ*_*ω*_, can be defined as follows^12^:


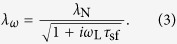


Here, *ω*_L_ is the Larmor frequency, given by *gμ*_B_*B*_⊥_/*ħ* where *g* is the Landé electron *g*-factor, *μ*_B_ is the Bohr magneton, *B*_⊥_ is the out-of-plane magnetic field and *ħ* is the reduced Plank constant and *τ*_sf_ is the spin relaxation time, given by 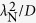 where *D* is the diffusion constant – taken to be to be 2.7 × 10^−3^ m^2^/s^13^.

For the identification of *λ*_N_ in straight wire lateral spin valves, there was no field applied out of plane and so the denominator in equation (3) goes to unity. By fitting this equation using least-squares fitting method *λ*_Cu_ is found to be (310 ± 30) nm at room temperature as shown in Fig. 1(b), in line with other previous works^8^,^9^,^10^,^13^,^14^,^15^.

**Figure 2:**
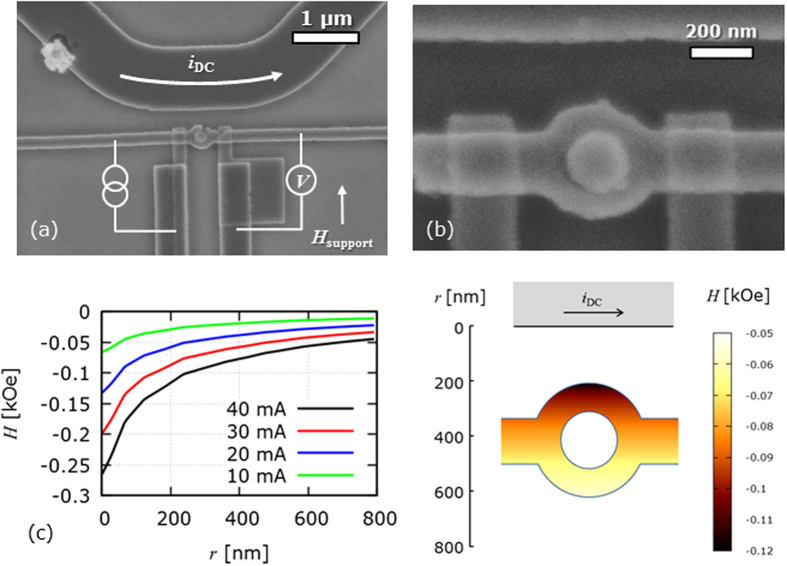
Spin-current operation. Scanning electron micrographs of spin operators showing (**a**) the current-carrying bar used to induce the Ampère field and the lateral spin-valve with a medial ring and (**b**) a close-up of the medial ring. Note the centre of the nano-ring is filled with resist (≈420 nm thick) due to the lift-off process. This did not affect the measurements as the resist thickness of 420 nm is much thicker than the Cu thickness of 70 nm (**c**) The variance of the Ampère field across the ring for various currents along with a colour map of the perpendicular field within the ring structure for a current of 40 mA applied to the current carrying bar.

Scanning electron microscope images of spin operator devices are shown in Fig. 2(a,b). The operation of the spin operators is in the same way as a conventional lateral spin-valve with a ring was patterned into the spin diffusion channel. The medial ring had an outer diameter of (365 ± 5) nm and an inner diameter of (195 ± 5) nm. The Copper was deposited to a nominal thickness of 70 nm as with the straight wire devices. In addition to the ring a micron-sized current carrying bar was fabricated above the ring as shown in Fig. 2(a). By applying a potential to the current-carrying bar an out-of-plane Ampère field was induced. The magnitude of the perpendicular field varied across the ring, inducing a field gradient along the *y*-axis in Fig. 2(c). This field gradient is integrated along the flow of the electrons and is accumulated as a phase change of the electron spin. The field from the bar was estimated using a finite element modelling package, FEMM^16^, where the wire was modelled as a ring with an inner diameter. The results from the simulation are shown in Fig. 2(c), where the outer edge of the ring is 200 nm and 600 nm and the centre of the ring is 410 nm from the edge of the current-carrying wire. These calculations show that these Ampère fields of a few 100 Oe is expected to be applied in the centre of the spin operator by flowing a few 10 mA in the current-carrying bar.

**Figure 3:**
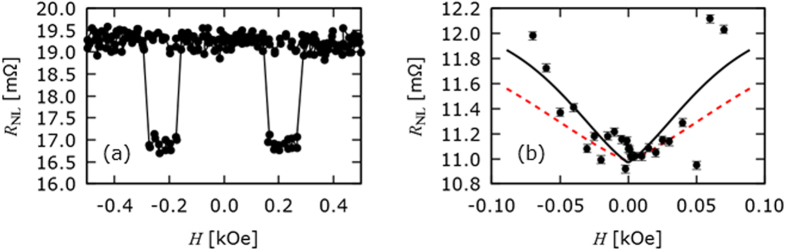
Spin-current operation in a ring device. (**a**) Typical non-local resistance measurement as a function of in-plane field for an lateral spin-valve with a medial ring, (**b**) The change in non-local resistance as a function of applied fields in the out-of-plane direction. The black line shows the least-squares fit of the analytical solution to Equation (4) to the data and the red dashed line shows the same fit using the parameters from nominally identical devices without a medial ring structure.

A typical non-local measurement for a lateral spin-valve with a medial ring is shown in Fig. 3(a). In order to evaluate the effect of the gradient of the field across the ring on the spin current, a saturating field (+2 kOe) was applied parallel to the ferromagnetic injector/detector pair. The magnetisation of the injector and detector pair was therefore set to be anti-parallel and the field was reduced to (−0.2 ± 0.05) kOe. This supporting field remained applied to the device to ensure that the magnetisation remained in the anti-parallel state. Measurements of the non-local voltage were taken with increasing current in the current carrying bar (see Fig. 3(b)).

The non-local resistance in the presence of a field in the out-of-plane direction is given by the following:


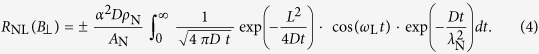


The solution to equation (4) provides the field dependence of *R*_NL_ and an analytical solution is provided in ref. 17. This equation was fitted to the data with the polarisation of the injector/detector wires and the spin-diffusion length as free fitting parameters. The path-length between the injector and detector (800 nm) was taken as the distance of the shortest path along the wire and around the centre of the ring. The value of the field applied in the out-of-plane directions was taken to be the value calculated in Fig. 2(c) at the centre of the ring. In this manner, the “effective” spin diffusion length can be extracted from the fit to the data. Figure 3(b) shows the data for the non-local resistance in the antiparallel injector/detector magnetic configuration and the fit of the solution to equation (4) to the data. For comparison, a calculated line (red) with spin-diffusion length of 310 nm is shown. For the ring device, the polarisations of the injector/detector, *α*, were found to be 3.6%. Over the samples that were measured in this and previous work the interfacial spin polarisation ranged by an order of magnitude from approximately 1 to 3%^18^. Therefore, we ignored any distributions in polarisations in our analysis.

The effective spin diffusion length, *λ*_S_, for the lateral spin-valve with a medial ring was calculated to be (410 ± 60) nm at room temperature using a least-squares fit (black line in Fig. 3(b). By comparing the black fit with the least-squares fit for the straight nano-wire (red dotted line in Fig. 3(c)), an increase of up to 30% as compared with the conventional lateral spin-valve. This increase was induced by the arithmetical summation of the pure spin currents flowing through the upper and lower paths of the nano-ring. These spin currents have different phases since they experienced different magnetic fields as illustrated in Fig. 2(c). We calculated the magnitude of this field to be 0.10 and 0.06 kOe for the middle the upper and lower paths respectively for a 40 mA current. For the sake of argument the field values in the centre of the ring are used to represent each dc current. It should be noted that the phase change would be a result of the integral of the field gradient along and perpendicular to the electron spin diffusion path. The spin-diffusion lengths of the two channels may be different due to local conductivity variations in the Cu nano-ring. Even so, the distributions should be small and could not be detected by the macroscopic non-local measurements. Hence, we used a single spin-diffusion length as a first approximation in our analysis to confirm the improvement to the efficiency of the device due to the ring.

**Figure 4:**
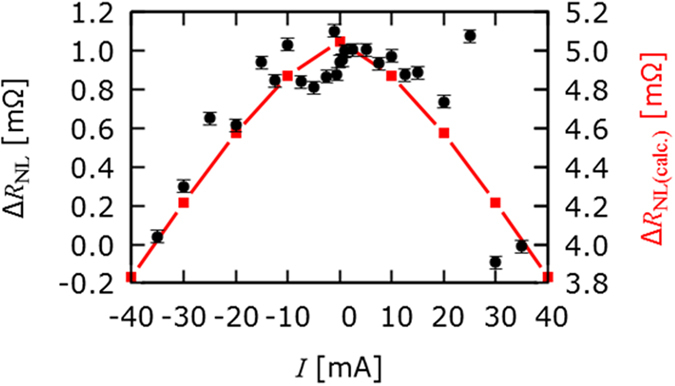
Arithmetical summation of the spin channels. Measured change in the non-local resistance (black circles, left hand vertical axis) and the calculated sum of the non-local signals in the upper and lower spin channels (red squares & lines, right hand vertical axis) as a function of the current applied to the current-carrying bar.

To confirm that this effect was due to the field gradient across the ring, and not simply due to the spin transport characteristics of the Cu, *i.e.* variation in the sample quality, the non-local voltage in the upper and lower sections of the ring was calculated using the analytical solution to equation (4). Our conclusion that the increase in the spin diffusion length is due to the arithmetical summation of the spin currents in the upper and lower channels is supported by the estimation of this value. The non-local resistance in the middle of the upper and lower channels was calculated using the solution to equation (4). The field values were taken from the FEMM calculation at the nominal position of the middle of the upper and lower channels (250 nm and 550 nm). The sum of these values (red squares and lines, right hand vertical axis) is shown in Fig. 4 alongside the experimentally observed values (black circles, left hand vertical axis). The magnitude of this estimation is approximately five times that of the experimental values. However, the estimation takes into account the values of the non-local resistance in the upper and lower channels alone and does not account for any further attenuation in the signal as the spin imbalance diffuses further along the spin channel and scatters into an equilibrium. It should also be noted that for a complete picture, the field should be integrated across the across the ring. However, it suffices as an estimate and supports our phenomenological observation that the increase in spin diffusion length is due to the arithmetical summation of the spin currents in the upper and lower paths of the ring in the spin diffusion channel.

## Discussion

The advantage of employing such a nano-ring configuration in a lateral spin-valve structure is that the oscillation period for the measured signal is almost half that for a straight wire. This shorter period is induced by the multiplication of the Hanle resistance caused by the recombination of the spin channels, which is very useful for future device applications as the power consumption of the operation of the gate can be minimised. Here, the signal amplitude can be further enhanced by replacing the Ohmic interface with the ballistic tunnelling one, especially for the spin injector^19^ improving a signal to noise ratio for the applications. Additionally, the results clearly demonstrate that spin-current operation is achieved based on the Larmor precession of the electron spins induced by the Hanle effect, which is suggested to be multiplied by the number of the regions under different perpendicular fields and to be divided by the number of the parallel current paths. This arithmetical operation of the spin current can form a basis of a spin logic circuit. It should be noted that such spin-current operation can only be achieved in the lateral spin-valve configurations with precise control in the dimensions.

In summary, we have successfully demonstrated spin-current modulation by a gate field introduction in a Cu nano-ring, which is observed to be much more effective than that induced by a simple Hanle effect. Our observation suggests the arithmetical operability of a spin current, which would be useful not only for future spin logic circuits but also for fundamental studies on intrinsic spin-current behaviour.

## Methods

### Device fabrication

The LSV devices were fabricated by conventional electron-beam lithography (JEOL, JBX-6300FS) and lift-off processes on a thermally oxidised Si substrate. Two Ni_0.8_Fe_0.2_ nanowires were designed to be 30 nm thick and 200 nm wide with different shapes at their ends (square and sharp) to induce a difference in their magnetisation-reversal fields. They were deposited using high vacuum (HV) sputtering (SP) system (Leybold, UNIVEX 350). After their lift-off, these wires were bridged by a Cu nanowire (70 nm thick and 200 nm wide) made by the same manner. The Cu was evaporated from a high purity source (99.9999%). Before the Cu deposition, the surfaces of the Ni_0.8_Fe_0.2_ wires were cleaned by Ar-ion milling (10 s at 50 W) to remove oxides and contamination. Electrical contacts to these wires were finally made by photolithography (EGV, Mask Aligner) and lift-off process after the deposition of Cr (10 nm)/Au (150 nm) layers using an electron-beam evaporator (Leybold, UNIVEX 350).

### Magnetotransport measurement

The transport properties of the LSVs were assessed by non-local magnetoresistance measurements with a “dc reversal” method^20^ using a Keithley 2400 current source and 2182a nanovoltmeter. An electrical current of 100 μA and 25 μA was used to measure the conventional lateral spin-valve and ring lateral spin-valve devices respectively. A global magnetic field was applied up to a maximum of ±2 kOe. All measurements were taken in air at room temperature.

## Additional Information

**How to cite this article**: Murphy, B. A. *et al.* Highly Efficient Spin-Current Operation in a Cu Nano-Ring. *Sci. Rep.* **6**, 37398; doi: 10.1038/srep37398 (2016).

**Publisher’s note:** Springer Nature remains neutral with regard to jurisdictional claims in published maps and institutional affiliations.
